# Reaching the Diagnosis of Checkpoint Inhibitor-Induced Diabetes Mellitus in Different Clinical Scenarios: A Real-World Application of Updated Diagnostic Criteria

**DOI:** 10.3390/diseases12020040

**Published:** 2024-02-14

**Authors:** Anna Angelousi, Dimitrios C. Ziogas, Vasiliki Siampanopoulou, Chrysoula Mytareli, Amalia Anastasopoulou, George Lyrarakis, Helen Gogas

**Affiliations:** 1 First Department of Internal Medicine, Unit of Endocrinology, Laikon Hospital, Center of Excellence of Endocrine Tumours (ERN), National and Kapodistrian University of Athens, 11527 Athens, Greece; v.siampanopoulou@gmail.com (V.S.); xrysam0804@gmail.com (C.M.); 2 First Department of Medicine, Laiko General Hospital, School of Medicine, National and Kapodistrian University of Athens, 11527 Athens, Greece; ziogasdc@gmail.com (D.C.Z.); amanastasop@yahoo.gr (A.A.); lyrarakis.g@hotmail.com (G.L.); helgogas@gmail.com (H.G.)

**Keywords:** immune checkpoint inhibitors, adverse events, autoimmune diabetes mellitus, anti-GAD, anti-IA2

## Abstract

Background: Checkpoint inhibitor (CPI)-associated diabetes mellitus (CPI-DM) is a rare immune-related adverse event (irAE) that presents with variable clinical manifestations. Data about its pathogenesis have not yet been adequately studied. Methods: Applying the recently updated diagnostic criteria from the American Diabetes Association, we retrospectively reviewed the medical records of all CPI-treated patients referred to our endocrinological unit for managing their endocrine irAEs and analyzed the incidence of CPI-DM, its clinical characteristics, and its management. Results: Among the 326 CPI-treated patients with endocrine irAEs, 4 patients met the updated criteria for the diagnosis of CPI-DM, representing 1.22% of all endocrine irAEs in our cohort. These four patients presented with distinct clinical scenarios regarding the irAE onset, the underlying malignancy, the administered CPI regimen, and the type of circulating autoantibodies. Conclusion: The variable presentation of CPI-DM and the non-standard sensitivity of the presence of the type 1 DM traditional autoantibodies highlight the need for distinct guidelines and increased awareness of its diagnosis and management.

## 1. Introduction

Checkpoint inhibitors (CPIs) have changed the management of several cancer types [1], leading to durable T-cell-mediated responses but at the price of a new array of immune-related adverse events (irAEs) [2]. IrAEs include a diverse range of inflammatory events affecting the skin (e.g., dermatitis); the gastrointestinal system (e.g., colitis); the liver (e.g., hepatitis); the entire endocrine system including the thyroid, the pituitary gland, the adrenals, the parathyroids, and the endocrine pancreas; as well as other less frequently encountered manifestations [3]. Among them, CPI-induced endocrinopathies have been reported in up to 40% of patients, with thyroid disorders being the most common [4,5]. As the indications of CPIs expand into more cancer types and adjuvant and neoadjuvant settings and patient survival improves, the prevalence of endocrine irAEs will correspondingly increase, and their spectrum will broaden to include more rare entities, such as primary adrenal insufficiency, central diabetes insipidus, primary hypoparathyroidism, lipodystrophy, osteoporosis, hypergonadotropic hypogonadism, or Cushing disease, which were recently presented by our group as well as diabetes mellitus (DM) [6].

Regarding CPI-DM, the available data are limited to case reports and small series that have applied heterogeneous diagnostic criteria [7]. New-onset hyperglycemia or the worsening of pre-existing DM during immunotherapy can be attributed to many etiologies, including exogenous glucocorticoid administration, immune-related pancreatitis, rapid autoimmune β-cell destruction, and lipodystrophy [8]. CPI-DM occurs in 0.2% to 1.4% of cancer patients receiving CPI-based regimens either as monotherapies or in combination with other agents (e.g., other CPIs, TKIs, or chemotherapies), and it has a more acute onset compared to classic type 1 DM [8,9,10,11,12,13,14,15]. Despite its rarity, CPI-DM is of significant clinical concern, with potentially life-threatening complications. Many cases, at the time of diagnosis, may present with diabetic ketoacidosis (DKA) or severe hyperglycemia grade 3–4, and others may maintain persistent insulin deficiency or experience the life-long risks of DM complications [16]. Thus, the early detection and management of CPI-DM are necessary to prevent significant morbidity and mortality [17].

Several efforts for a better characterization of CPI-DM have been made to aid clinicians [8]. In a recent systematic review, Wu et al. [7] updated the diagnostic criteria for CPI-DM, as follows: (i) evidence of new-onset hyperglycemia post-CPI treatment in the diabetes range according to the classical American Diabetes Association criteria (random blood glucose ≥ 11.1 mmol/L or HbA1c ≥ 6.5%) [18], and (ii) insulin deficiency determined by low C-peptide (<0.4 nmol/L or 1.2 ng/mL) at presentation and/or DKA, without known treatment with a sodium-glucose co-transporter 2 (SGLT2) inhibitor, in patients who have previously received CPIs. Therefore, based on the subsequently mentioned criteria, we provide our experience regarding the identification and management of CPI-DM cases at our site and discuss some upcoming considerations to identify opportunities for further quality improvement in patient care.

## 2. Materials and Methods

Between 2018 and 2023, we retrospectively reviewed the medical records of all patients who were referred to the Endocrinology Department of our tertiary university-affiliated hospital (Laiko General Hospital, Athens, Greece) for the management of various endocrine irAEs in order to identify those with CPI-DM. For the confirmation of CPI-DM diagnosis in our cohort, we followed the recently proposed criteria [7]. All patients were tested for anti-islet cell autoantibodies, such as anti-glutamic acid decarboxylase (anti-GAD), anti-tyrosine phosphatase-like insulinoma antigen 2 (anti-IA2), and anti-zinc transporter protein 8 (anti-ZnT8). Medical files were retrospectively retrieved, and all demographic, clinical, radiographic, histopathological as well as treatment data were recorded for each patient. Telephone follow-up was also performed when needed. The grading of irAEs was based on the National Cancer Institute Common Terminology Criteria for Adverse Events (version 5.0). Tumor staging was characterized according to the 8th edition of the American Joint Committee on Cancer staging system before CPI treatment. CPI treatment response was assessed as complete response (CR), partial response (PR), stable disease (SD), or progressive disease (PD) based on imaging follow-up from the beginning of immunotherapy. In accordance with the ethical declaration of Helsinki (1964) and its later amendments, all presented patients gave written informed consent for this publication on a local form approved by the Medical Ethics Committee of our institution prior to reporting their cases.

## 3. Results

**Figure 1:**
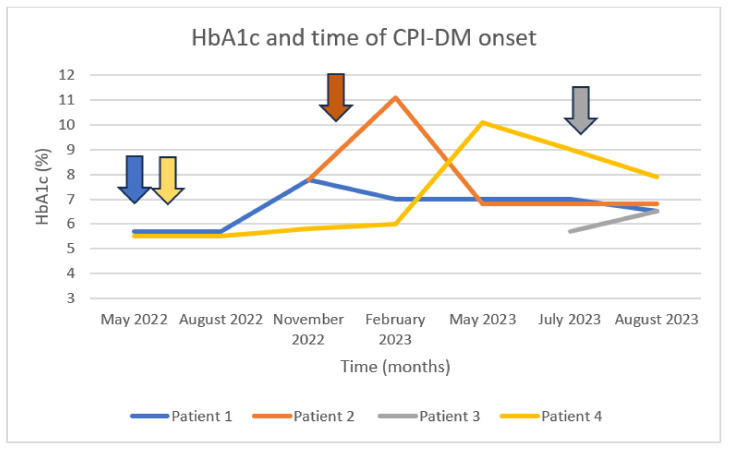
HbA1c levels at the time of CPI-DM diagnosis. Patient 1 treated with nivolumab and ipilimumab since May 2022, presenting inadequate glycemic control 5 months after the initiation of the combination treatment. Patient 2 treated with pembrolizumab since January 2023, presenting with elevated glucose levels one month after its initiation. Patient 3 treated with nivolumab in combination with oncolytic virus RP3; she was hospitalized due to DKA after the first administration of nivolumab (anti-PD-1). Patient 4 treated with pembrolizumab since May 2022, hospitalized due to DKA 12 months after the initiation of the anti-PD-1 treatment. The arrows represent the initiation of CPI-based treatment.

During the examined period, 1077 patients were treated with CPI-based regimens at our site, and 326 of them were referred to our endocrinology department. Four patients—2 males and 2 females—met the updated criteria for the diagnosis of CPI-DM, representing 1.22% of all endocrine irAEs detected in our cohort. A total of 3 out of 4 patients were treated with an anti-PD-1 agent, and the 4th patient was treated with a combination of an anti-PD-1 and an anti-CTLA-4 CPI. One patient presented with severe (grade 4) CPI-DM, being enrolled in an open-label, multicentered, phase I study (NCT04735978) of the oncolytic virus RP3 in combination with nivolumab in previously multi-treated patients with solid tumors. Table 1 summarizes the data of these 4 cases and gives details about their underlying malignancy, their oncological treatment, and their laboratories at the time of diagnosis of their immune-related endocrinopathy. Figure 1 depicts the fluctuation in HbA1c levels for all these cases up to the onset of CPI-DM and the subsequent normalization.

### 3.1. Clinical Scenario 1: Anti-IA2 Positivity

A 75-year-old female with a past medical history of DM type 2 since 2017, under treatment with oral antidiabetic drugs (850 mg of metformin once daily) and adequate glycemic control (HbA1c: 5.7%), was diagnosed with a subcutaneous melanoma lesion on her left thigh with an unknown primary site (probably a satellite or in-transit lesion of a regressed initial melanoma), stage TxN1cM0 (*AJCC 8th edition*), BRAF wild-type, in February 2018. She successfully completed one year of adjuvant immunotherapy with nivolumab (240 mg every 2 weeks), but after 20 months, she experienced a local melanoma relapse that was resected again, and she restarted treatment with nivolumab plus ipilimumab (a combination of PD-1 with anti-CTLA-4). The latter scheme was interrupted after 3 months because of CPI-induced glomerular nephritis (grade 3), treated with methylprednisolone for a total period of 3 weeks without any severe deterioration of her glycemic control, and after the resolution of irAE to grade 1, a new combinatorial regimen with pembrolizumab plus lenvatinib (an anti-PD-1 plus a pan-tyrosine kinase inhibitor, TKI) was initiated. Five months post-nivolumab/ipilimumab discontinuation and under treatment with pembrolizumab/lenvatinib, she presented with poor glycemic control on self-monitoring (glucose stick > 200 mg/dL), confirmed by elevated HbA1c levels at 7.8%. The measurement of C-peptide was within a normal range (3.15 ng/mL; normal range: 0.5–7.19 ng/mL); however, anti-IA2 levels were positive (141.82 IU/mL; normal: <20 IU/mL), and anti-GAD levels were negative (1.83 IU/mL; normal, <30 IU/mL). Insulin therapy (insulin glargine) was initiated as a monotherapy, progressively increasing from 6 ui to 17 ui/day. Six months after the initiation of insulin therapy, she presented with normal glycemic control with a new Hb1Ac of 7%. No other ir endocrinopathy has been diagnosed so far. The combination treatment of pembrolizumab and lenvatinib was not interrupted, and the patient continues to receive it requiring, though, increasing doses of insulin glargine (currently as a monotherapy).

### 3.2. Clinical Scenario 2: Anti-GAD Positivity

A 57-year-old male with known and well-controlled DM since 2020 with oral antidiabetic medication (850 mg of metformin twice daily, and Hb1Ac < 6.0%), and a positive family history for DM (his mother was under treatment for DM type 2), was diagnosed with stage IIB melanoma (*AJCC 8th edition*) of his right thorax left (Breslow thickness, 4 mm, with negative SLN, T3bN0M0) in May 2022. He started adjuvant treatment with pembrolizumab in January 2023. One month after the initiation of anti-PD-1 inhibition, the patient was referred to the endocrinology department because of poorly controlled glucose levels, with elevated morning pre-prandial glucose levels (>300 mg/dL) and increased HbA1c at 11.1%. Endocrinology work-up showed low C-peptide levels (0.31 ng/mL; normal range: 0.7–5.19 ng/mL), positive anti-GAD (141 IU/mL; normal: <17 IU/mL), and negative anti-IA2 autoantibodies (7.3 IU/mL; normal: <20 IU/mL). Insulin therapy (scheme with insulin degludec and insulin aspart) was initiated, and the patient presented with adequate glycemic control in the first three months (HbA1c = 6.8%). It should be noted that in April 2023, the patient also presented with clinical symptoms of isolated corticotrope deficiency (low cortisol = 0.5 μg/dL; normal range: 4.8–19.46 μg/dL), with inappropriately normal ACTH (23.1 pg/mL; normal range: 6–48 pg/mL); adrenal autoantibodies were negative (<1/10). A replacement treatment with oral hydrocortisone (30 mg daily) was then initiated. The rest of the pituitary hormone function was intact. The patient did not resume adjuvant pembrolizumab, with no evidence of disease to date. However, during the 3-month follow-up, he was still on insulin treatment (degludec and aspart).

### 3.3. Clinical Scenario 3: DKA Manifestation with Positive Anti-GAD, Anti-IA2, and Anti-ZnT8

The most recently added patient in our series is a 54-year-old female diagnosed with adenocarcinoma of the right colon in 2019, with T2N0 colorectal cancer (KRAS mutated, NRAS wild-type, BRAF wild-type, MSI low) and treated surgically with right hemicolectomy followed by active surveillance. At the time of the first follow-up, she presented with two liver metastases; thus, she started first-line chemotherapy with FOLFOXIRI and bevacizumab for 6 months. Next, the patient underwent debulking surgery, including hepatic metastasectomy and local ablation, and continued with FOLFOXIRI and bevacizumab for 6 months when she presented with chemotherapy-induced thrombocytopenia. After the recovery of platelet count, she was enrolled in an open-label, multicenter, phase I study (NCT04735978) of the oncolytic virus RP3 in combination with nivolumab in previously multi-treated patients with solid tumors. She received four oncolytic intralesional infusions with no complications, but after the first nivolumab administration, the patient was referred to the emergency department for extreme fatigue, polydipsia, and polyurea. The laboratory results revealed DKA (Glucose = 674, pH = 6.9, low C-peptide = 0.72 ng/mL, and slightly elevated HbA1c = 6.4%). Anti-islet autoantibody measurements were positive: anti-GAD, 42.7 U/mL (<1); anti-IA2, 18.5 U/mL (<1); and anti-ZnT8, 18.6 U/mL (<15). The patient had no previous personal history of diabetes; however, her son was diagnosed with type 1 DM at the age of 5 years old, and her mother was diagnosed with type 2 DM at the age of 60 years. She was treated according to the protocol of DKA [19], and she was discharged from the hospital with a basal/bolus scheme of insulin therapy (insulin glargine and insulin lispro). During her hospitalization, she was additionally diagnosed with overt subacute thyroiditis, and a short-term treatment with corticosteroids was also initiated. The patient discontinued the protocol treatment due to grade 4 AE with stable disease, according to cancer assessment. During the first month of follow-up, she presented with adequate glycemic control on insulin therapy.

### 3.4. Clinical Scenario 4: DKA Manifestation with No Antibody-Based Confirmation of CPI-DM

A 69-year-old male with no past medical history of DM was diagnosed with localized renal cancer in 2018, for which he underwent a partial right nephrectomy and received no adjuvant treatment. His disease relapsed in February 2022 with a new local lesion, and after a complete right nephrectomy, he consequently started adjuvant pembrolizumab in May 2022. After almost one year of anti-PD-L1-treatment, he was admitted to our hospital with symptoms of extreme fatigue, polyuria, and polydipsia. Blood analyses revealed DKA (glucose ≥ 300, pH = 7.18), low C-peptide levels (0.93 ng/mL; normal range: 0.7–5.19 ng/mL), and Hb1Ac levels at 9.7%. Anti-islet autoantibodies were found to be negative. To control his DKA, he received a basal–bolus insulin therapy scheme with insulin glargine and lispro. Experiencing a grade 4 irAE and having almost completed his adjuvant CPI treatment, he never resumed his adjuvant immunotherapy. During the 4-month follow-up, the patient presented with improved glycemic control (HbA1c = 7.9%), with the same insulin therapy scheme, without presenting with any other endocrine ir complications so far.

## 4. Discussion

CPI-DM is a rare endocrine irAE, but more and more patients have been labeled with this diagnosis. Up until recently, no clear diagnostic criteria existed; however, a recent systematic review provided some more specific characteristics of CPI-DM to delineate this disorder from type 1 DM [7]. Thus, based on these criteria, we tried to identify the incidence of CPI-DM and discuss the clinical challenges in the diagnosis and management of patients with CPI-DM in a real-world setting. Running through our medical records, we recognized only four patients meeting the updated diagnostic criteria for CPI-DM, representing 1.22% of all endocrine irAEs recorded in our population. We should consider this incidence more representative since the endocrinology assessment for the rest of the CPI-treated patients was away from our site, and such a rare ir endocrinopathy may be misdiagnosed.

Next, we will debate the findings of our small series with the current evidence regarding CPI-DM. In our series, all four patients experiencing CPI-DM received anti-PD-1 agents, and one of them was in combination with an anti-CTLA-4 agent. Indeed, recent data [20] have demonstrated that anti-PD-1 and anti-PD-L1 agents are more prone to trigger the onset of DM in comparison to anti-CTLA-4. Moreover, and in agreement with a systematic review by Wu et al. [7], including 192 confirmed CPI-DM cases (the time of diagnosis: from 6 to 24 weeks after the onset of immunotherapy, with DKA in 69.7% of cases), the time of diagnosis in our small series ranged from 1 month to 1 year, while 2 of the 4 cases presented with DKA. According to Stamatouli et al. [10], CPI-DM presented with DKA in 59% of cases in the peridiagnosis period, whereas only 5.2% of them reported a previous history of pre-existing DM type 2. However, two out of our four patients had a known past medical history of DM type 2, treated adequately with an oral antidiabetic regimen (metformin) but presented with further deterioration of glycemic control, developing rapidly new insulin dependence after immunotherapy initiation. In the majority of recorded cases, fasting C-peptide levels were low at presentation with CPI-DM [7,10], as in three of our cases. 

In a large case series including 27 patients that developed DM following treatment with anti-PD-1 or anti-PD-L1, the measurement of antibodies (anti-GAD, anti-ZnT8, anti-IA2, and islet cell antibodies) associated with autoimmune DM was performed in 25 of them. A control group of 12 patients with similar cancer types and treatment regimens with no DM was also analyzed [10]. At least one antibody was positive in 40.4% (10/25) of the cohort, compared to 25% (3/12) of the control group [10]. The vast majority (91.7%) presented with seropositivity for the anti-GAD autoantibody, either alone or in combination with other islet autoantibodies [21]. Moreover, in the same study, the measurements of autoantibodies before and during CPI treatment were conducted in only three patients [10]. Lo Preiato et al. [22] agreed that a total of 43.0% of patients had positive anti-GAD antibodies in their database, in contrast with the much higher prevalence of type 1 DM, with no comparison with a control group. Many reports have identified CPI-DM cases with both positive and negative specific autoantibodies but without strong conclusions [23,24,25,26]. A case report and literature review study showed that islet autoantibodies may be present before CPI treatment in non-diabetic patients and, when exposed to CPI therapy, may precipitate the onset of DM [27]. Positive autoantibodies were associated with earlier and more severe presentations of CPI-DM [23,24,25,26]. In our series, positive islet autoantibodies were detected in three of four cases (one with anti-IA2, one with anti-GAD positivity, and one with anti-IA2, anti-GAD, and anti-ZnT8 positivity). In a review of Wu et al. [7], lipase was elevated in 69.4% of cases with CPI-DM, and in a multicenter study by the German Dermato-oncology Group, 55% of patients (12/22) with newly diagnosed CPI-DM also had lipase elevation shortly before or after their diagnosis of diabetes [28], suggesting a possible involvement of the exocrine pancreas. In a nested case-control study of 8 CPI-DM cases and 16 controls, C-peptide levels, islet autoantibodies, and pancreatic enzymes were prospectively measured in blood serum, and changes in their levels before and after CPI-DM onset were comparable to patients without ir DM [29]. Moreover, the presence of positive islet autoantibodies in 1 out of 16 asymptomatic controls suggests that DM may pre-exist subclinically and CPIs trigger its clinical manifestation [29]. Moreover, the incidence of pancreatitis in patients receiving CPIs is low, reaching up to 3% in patients treated with anti-CTLA-4; up to 1.6% in patients treated with anti-PD-1; and up to 2.1% in patients treated with a combination of anti-CTLA-4 and anti-PD-1, although the incidence of elevated amylase and lipase is much higher. Therefore, routine monitoring of pancreatic enzymes is not recommended, unless pancreatitis is suspected clinically [30]. In our case series, all patients had normal amylase levels, except for the first one, who had slightly increased amylase but with no clinical signs of pancreatitis.

The exact pathophysiology of CPI-DM has not been completely elucidated. CPI-DM presents similarities with DM type I, as both entities result from humoral CPI-DM and cellular immune-mediated β-cell destruction leading to insulin deficiency. The immunohistopathological analysis of pancreatic tissue from patients with CPI-DM showed infiltration of islets with macrophages and T-lymphocytes, with a predominance of cytotoxic CD8^+^ T-lymphocytes, a decrease in b-cell area, and an increase in a-cell area compared to patients treated with CPI without presenting with DM and euglycemic controls, indicating selective targeting of β-cells [31]. Islet-antigen-specific CD8^+^ T-lymphocytes have also been identified in the peripheral blood of patients with CPI-DM [32]. However, CPI-DM has several distinct characteristics. DM type 1 is a progressive disorder that is initially asymptomatic, although islet autoantibodies can be positive months to years before the development of overt hyperglycemia [18]. On the contrary, the destruction of β-cells appears to be more rapid in patients with CPI-induced DM, considering the fulminant presentation in most cases, as well as the early low or undetectable C-peptide levels [7]. Additionally, the ‘’honeymoon’’ period, which is associated with the partial recovery of β-cells after the clinical onset of DM type I, is absent in CPI-DM [8]. As previously reported, specific islet autoantibodies are inconsistent in the setting of CPI-DM, with a prevalence of approximately 50% in contrast to a prevalence of 90% in patients with DM type I [7]. CPI-DM may also coexist with other irAEs, for example, in 43.8% of 192 identified cases based on a study by Wu et al. [7]. Among the co-existing endocrinopathies, thyroid dysfunction is the most common one (20.8%), leading to a diagnosis of autoimmune polyendocrine syndrome type 2 (APS-2) [10]. Thus, in patients with a newly diagnosed ir endocrinopathy, a more aggressive screening strategy for other endocrinopathies should be followed [5]. 

Recently, several biomarkers have been identified that could predict the presentation of autoimmune DM in CPI-treated patients. A large recent study, which included a Japanese population, demonstrated a significant association of specific human leukocyte antigens (HLA) (HLA-DPA1*02:02 and DPB1*05:01 alleles and HLA-DPA1*02:02-DPB1*05:01 haplotypes) with the risk of developing CPI-DM [33]. Stamatouli et al. [10] concluded that US patients with HLA-DR4 were at an increased risk of developing CPI-DM compared to US Caucasians and patients with DM type I. Many other reports have mentioned a correlation between the presence of HLA-DRB1*04:05 and HLA-DQB1*04:01 with CPI-DM [34,35,36,37], but more data are needed for strong conclusions. RNA and whole exome sequencing in tumor samples from CPI-DM patients showed no differences in the expression of conventional DM type I autoantigens compared with controls treated with the same CPIs for the same cancer types. However, a missense mutation in *NLRC5* (NOD-like receptor family CARD domain containing 5) was observed in 9 of the 13 CPI-DM patients but not in the control group [38]. Interestingly, all *NLRC5* mutations were germline, and their prevalence was significantly greater in CPI-DM patients compared to the general population (*p* = 5.98 × 10^−6^) [38]. Moreover, these germline pathogenic *NLRC5* mutations were absent from DM type I patients, suggesting that different pathophysiological mechanisms and molecular pathways are involved in the pathogenesis of CPI-induced DM compared to the autoimmune insulin-dependent type 1 DM. In addition, neutrophil counts, neutrophil–lymphocyte ratios, and neutrophil–eosinophil ratios in peripheral blood were increased, and the absolute lymphocyte and eosinophil counts were decreased at the onset of CPI-DM, as compared with 6 weeks prior, suggesting an autoimmune origin of this toxicity [33]. 

The recently updated European guidelines for the diagnosis and management of ir endocrinopathies propose screening with baseline measurement as well as regular monitoring of glucose levels every 4–6 weeks [5]. The HbA1c measurement might be misleadingly low due to the fulminant nature of diabetes onset [5]. Evidence of insulin deficiency (low C-peptide or DKA at presentation) is a key criterion for the diagnosis of CPI-DM [7]. Complementary analyses should include the assessment of exocrine pancreatic function (lipase levels), the exclusion of autoimmune lipodystrophy (serum triglycerides), and seropositivity as supportive criteria (islet autoantibodies) [7]. C-peptide testing can be repeated after 1 month to confirm the diagnosis and guide ongoing treatment [7]. Once the diagnosis of CPI-DM is confirmed, a multiple insulin injection regimen is recommended as the first-line treatment. CPI-DM is considered permanent, requiring lifelong insulin replacement, but a single case of remission has also been described in the literature [39]. Glucocorticoids are not recommended due to their uncertain efficacy and potential worsening of hyperglycemia [5]. In the course of the disease, a screening schedule for long-term DM complications should be set [5]. However, the development of CPI-DM does not prompt discontinuing CPI treatment [5].

## 5. Conclusions

In conclusion, CPI-DM is a rare but potentially life-threatening clinical entity, especially when presented with DKA. This autoimmune, insulin-dependent diabetes has similarities and differences compared with type 1 DM. Based on the updated diagnostic criteria, anti-islet autoantibodies and C-peptide levels should be measured to establish its diagnosis, without delaying insulin treatment. The application of these proposed criteria in a real-world population of CPI-treated patients gives a representative picture of different clinical scenarios with CPI-DM, increases the awareness of clinicians, and helps to further improve the diagnostic approach and care of these patients.

## Figures and Tables

**Table 1:** Clinical characteristics of cancer patients’ developed CPI-DM.

	Case 1	Case 2	Case 3	Case 4
Age (years)/gender (F/M)	75/F	57/M	54/F	69/M
Underlying cancer	Metastatic melanoma	Melanoma	Metastatic CRC	Renal cancer
Type of CPI	Nivolumab + ipilimumab	Pembrolizumab	Nivolumab plus + OV	Pembrolizumab
Previous history of DM	Yes	Yes	No	No
Family history of DM	Yes	Yes	Yes	No
BMI (kg/m^2^)	32.2	29.1	23.4	27.7
Onset of autoimmune DM presentation	5 months	1 month	1 month	12 months
DKA	No	No	Yes	Yes
Baseline glucose levels (mg/dL)	>200	>300	>300	>300
HbA1c (%)	7.8	11.1	6.4	9.7
C-peptide (0.7–5.19 ng/mL)	3.15	0.31	0.72	0.93
Islet autoantibodies	(+) Anti-IA2	(+) Anti-GAD	(+) Anti-IA2, (+) anti-GAD, (+) anti-ZnT8	Negative
Amylase (28–100 U/L)	135	49	68	36
Triglycerides (50–150 ng/dL)	110	156	249	73
Concurrent ir endocrinopathies	No	Hypophysitis (corticotrope axis deficiency)	Subacute thyroiditis	No
Follow-up since CPI-DM presentation (months)	6 months	3 months	1 month	1 month
Outcome	Insulin therapy–glycemic control HbA1c = 7%	Insulin therapy–glycemic control HbA1c = 6.8%	Insulin therapy–glycemic control HbA1c = 6.2%	Insulin therapy–glycemic control Hb1Ac = 9.3%

Abbreviations: CRC: colorectal carcinoma, CPI: checkpoint inhibitors, DM: diabetes mellitus, BMI: body mass index, HbA1C: hemoglobin A1C, anti-GAD: antibodies to glutamic acid decarboxylase, anti-IA2: antibodies to islet tyrosine phosphatase 2, anti-ZnT8: antibodies to zinc transporter 8.

## Data Availability

Data supporting this article are included within the reference list. Please contact the corresponding author for any further information.
